# Headaches, Heart Block, and a Heart Tumor: Unusual Presentation of Malignant Melanoma

**DOI:** 10.7759/cureus.80235

**Published:** 2025-03-07

**Authors:** Sahar Sohrabian, Ramona Mehrinfar-Zadeh

**Affiliations:** 1 Cardiology, University of California, Los Angeles, Los Angeles, USA

**Keywords:** cardiac tumor, heart block, malignant melanoma, metastatic tumor to the heart, right atrial cardiac mass

## Abstract

Cardiac tumors may range from benign primary cardiac masses to malignant metastatic tumors. The constellation of systemic symptoms and presenting findings in metastatic cardiac tumors may be non-specific and may lead to non-cardiac testing before the diagnosis is confirmed with advanced cardiac imaging. We present a case of malignant melanoma with metastatic disease to the heart presenting with headaches, altered mental status, and complete heart block. A detailed history and dermatologic exam should be pursued in any case of cardiac tumor, as metastatic melanoma has a predilection for cardiac involvement. The prognosis remains poor independent of chemotherapy, immunotherapy, and surgical resection, especially with a higher burden of metastatic disease.

## Introduction

Cutaneous melanoma represents one of the most aggressive skin cancers, with most cases due to exposure to ultraviolet light [1]. Early detection and surgical therapy remain the mainstay of treatment of local disease. However, if left unchecked, malignant melanoma may invade vascular and lymphatic beds, leading to hematogenous spread [2]. Malignant melanoma may rapidly spread to various organs with a predilection for the heart [3]. Most cases of metastatic melanoma to the heart are discovered post-mortem in asymptomatic individuals. However, the clinical presentation may include heart failure, pericardial effusion, cardioembolic sequalae, valve dysfunction, and arrhythmias. Diagnosis requires a detailed history and skin exam coupled with advanced cardiac imaging, including cardiac magnetic resonance imaging (CMRI) and positron-emission-tomography (PET). However, non-specific systemic symptoms may lead to an initial rheumatological and infectious work-up. In this report, we describe a case of cardiac metastatic melanoma presenting with acute stroke and complete heart block.

## Case presentation

A 78-year-old female with no past medical or surgical history presented to the emergency room with two weeks of progressive headaches, malaise, palpitations, and weakness. She noted vague back, neck, and jaw pain and weakness throughout her body associated with a 20-pound unintentional weight loss over the past two months but denied night sweats or poor appetite. The patient also noted slight hearing and vision changes that she attributed to her headaches since the onset of symptoms. She was not on medications at home, nor did she smoke, drink alcohol, or use recreational drugs. She lived with her two daughters and had no toxic exposure. 

On presentation, her vital signs showed a blood pressure of 165/70, a heart rate of 35 beats-per-minute, and oxygen saturation of 97% on room air. She was afebrile. Her exam was notable for 4/5 right lower extremity strength, musculoskeletal tenderness of the shoulders, decreased vision in the left eye, and decreased hearing on the left, which the patient noted to be progressive since the onset of her symptoms. Her cardiovascular exam was notable for an irregular and sometimes slow pulse alternating with a normal regular pulse. There were no murmurs, rubs, or gallops, and peripheral pulses were robust. 

Blood laboratories showed normal blood cell counts with normal renal, liver, and thyroid function and electrolyte levels. Her serum inflammatory markers were elevated, but additional rheumatological labs were normal (Table 1). 

**Table 1:** Blood laboratory values WBC = white blood cell count; RBC = red blood cell count; MCV = mean corpuscular volume; MCH = mean corpuscular hemoglobin; MCHC = mean corpuscular hemoglobin concentration; RDW = red cell distribution width; MPV = mean platelet volume; TSH = thyroid stimulating hormone; IFA = indirect immunofluorescence assay; dsDNA = double-stranded deoxyribonucleic acid; RNP = ribonucleoprotein; EIA = enzyme immunoassay; Ab = antibody

Laboratory	Reference	Result
WBC	4.00-11.00 1000/UL	9.71
RBC	3.67-5.11 MILL/UL	5.09
Hemoglobin	11.6-15.4 g/dL	15.4
Hematocrit	34.3-45.4%	44.7
MCV	80.0-100.0 FL	85.8
MCH	27.0-33.0 pg	28.3
MCHC	32.0-36.0 g/dL	33
RDW	11.7-14.4%	12.9
Platelet count	150-450 1000/UL	438
MPV	9.4-12.3 FL	10.4
Glucose	70-99 mg/dL	126
Sodium	136-145 mmol/L	139
Potassium	3.5-5.0 mmol/L	3.8
Chloride	98-107 mmol/L	102
Carbon dioxide	22-31 mmol/L	26
Anion gap	10-20 meq/L	14
Urea nitrogen	9.8-20.1 mg/dL	11.9
Creatinine	0.57-1.11 mg/dL	0.84
Calcium	8.4-10.2 mg/dL	9.2
Sedimentation rate (ESR)	<30 mm/hr	53
C-reactive protein	<5.1 mg/L	11.5
TSH	0.39-4.60 mIU/L	1.59
Antinuclear antibody, IFA	<40 titer	<40
dsDNA antibody, IFA	<10 titer	<10
Smith (SM) Ab (EIA)	<20 units	1
RNP antibody	<20 units	3
SSA (Ro) Ab (EIA)	<20 units	1
SSB (La) Ab (EIA)	<20 units	1
SCL-70 Ab (EIA)	<20 units	2

**Figure 1:**
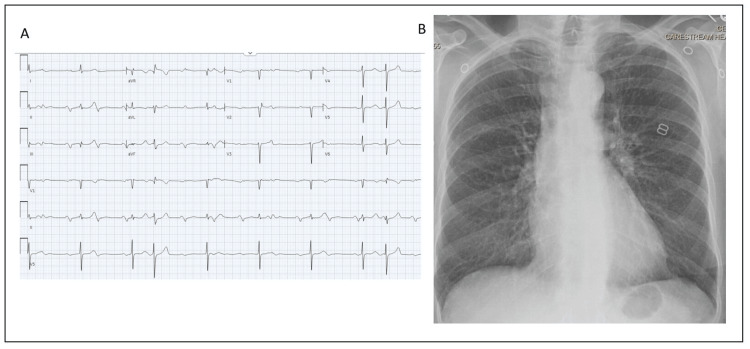
Initial diagnostic studies A – Electrocardiogram showing sinus/ectopic atrial rhythm with intermittent complete heart block and junctional escape rhythm. B – Chest radiograph without abnormalities.

Her electrocardiogram (ECG) is shown in Figure 1. The chest radiograph was unremarkable. A CT scan of the brain revealed acute L frontal and temporal lobe infarcts, suspicious for embolic phenomenon to large vessel territories. The results of transthoracic echocardiography revealed normal cardiac function with no valvular abnormalities. She was admitted for further work-up, and given her visual disturbance, an eye exam was performed, which revealed left optic disk edema suggestive of arteritic ischemic optic neuropathy. Although the patient denied jaw claudication or scalp tenderness, a temporal artery biopsy was negative for giant cell arteritis.

**Figure 2:**
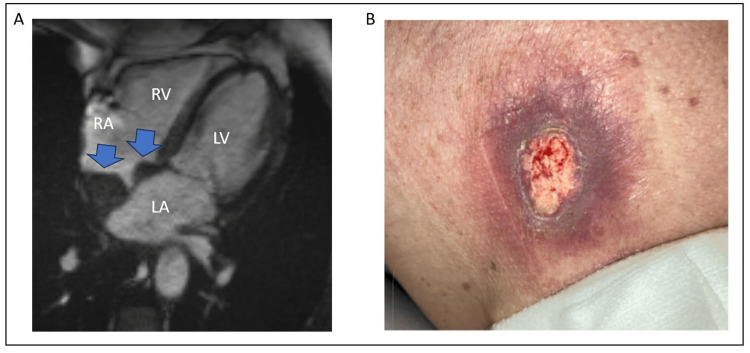
Cardiac MRI and skin lesion A – Cardiac MRI showing a large mass extending across the inter-atrial septum (blue arrows). B – Ulcerating skin lesion on the patient’s back. RA = right atrium; LA = left atrium; RV = right ventricle; LV = left ventricle.

The patient became progressively encephalopathic over the next few days. Cerebrospinal fluid analysis after lumbar puncture was limited due to minimal fluid volume retrieval and scant cells but was not consistent with infectious encephalitis. During magnetic resonance examination of the great vessels for vasculitis, a large intra-atrial cardiac mass was noted, protruding into the right atrium (Figure 2A). This mass had a heterogenous pattern of late gadolinium enhancement (LGE) consistent with malignancy rather than atrial thrombus. Based on the added history obtained from the family, the skin on the patient’s back was examined and revealed an ulcerating skin wound (Figure 2B). Biopsy of this skin lesion confirmed ulcerating advanced melanoma.

**Figure 3:**
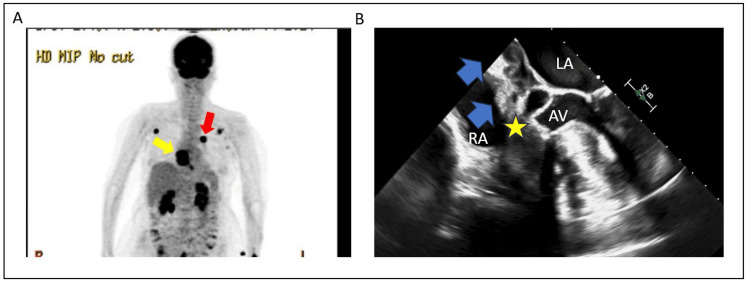
Advanced cardiac imaging A – PET/CT whole body scan showing enhancement of suspected primary lesion on the patient’s back (red arrow) and in the heart at the level of the atria (yellow arrow). B – Transesophageal echocardiogram showing extension of tumor across the inter-atrial septum (blue arrows) with encroachment of the aortic valve. The yellow star denotes the approximate anatomical location of the compact AV node. PET/CT = positron emission tomography/computed tomography; AV = aortic valve; all other abbreviations as per Figure 2.

A positron emission tomography CT showed intense fluorodeoxyglucose (FDG) uptake in the skin lesion, multiple lymph nodes in the chest, and the cardiac intra-atrial mass (Figure 3A). She continued to experience episodes of complete heart block and underwent leadless pacemaker placement with concomitant trans-esophageal echocardiography (TEE) (Figure 3B) to better delineate the mass for potential surgery. Plans were made for urgent chemo- and immunotherapy, but she had rapid progressive clinical decline over the next three weeks and passed away under palliative care. Given the constellation of findings and results of testing, she was diagnosed with metastatic malignant melanoma with cardiac involvement, thromboembolic stroke, and likely leptomeningeal seeding, given progressive obtundation.

## Discussion

Cardiac masses are more likely to be metastatic disease rather than primary cardiac tumors [3]. Due to anatomical proximity and lymphatic drainage, most cardiac metastases are from thoracic tumors, including bronchogenic and breast carcinomas with a predilection for the pericardium [4].

Metastatic melanoma is a rare disease, but metastatic spread is common in malignant melanoma [5]. The most common organs affected by metastatic melanoma are the lungs, brain, liver, and bone. In a study of over 200 patients with first presentation of melanoma, almost one-third of patients had metastatic disease (regional lymph node or organ involvement) [2]. However, of all cardiac metastatic diseases, malignant melanoma is second only to mesothelioma as the primary malignancy [4]. Metastatic disease at presentation or at follow-up was more likely with ulcerated skin melanoma than non-ulcerated lesions. Melanoma has a disproportionately higher incidence of cardiac metastases compared to more common cancers, such as lung and breast. The exact mechanisms of this predilection for the heart are unknown but thought to be due to a high affinity for vascular structures and specifically an ability to adhere to vascular endothelium, making diffuse spread along the vena cava more likely. There is not only a predilection for the heart but specifically for the right atrium, which is in line with the vena cava [5]. 

Most patients do not have cardiovascular symptoms, and cardiac metastasis is often found post-mortem. The presence of symptoms may depend on the size and site of the metastatic mass. The most common symptom is shortness of breath, and the most common chamber for metastasis is the right atrium [6]. Systemic symptoms, including weight loss and fevers, may be non-specific. Mass effects resulting in cardiac symptoms and thromboembolic events may occur in combination or in isolation with cardiac metastatic disease. In the case presented, the PET-CT findings and TEE images delineate the course of the mass encroaching on the non-coronary leaflet of the aortic valve. This anatomical area is adjacent to the compact atrio-ventricular node that may be compromised by an encroaching mass, resulting in conduction disturbances, which is a rare finding for cardiac malignancies. Conduction system disturbances in the setting of cardiac tumors are rare and have been reported only in case reports. Most cases have described advanced stage metastatic primary lung carcinoma presenting with large cardiac masses and multi-organ spread [7,8]. Patients with a prior history of malignant melanoma may present with cardiac metastatic disease many years after diagnosis and treatment [9,10]. Cheng et al. describe a case where elective permanent pacemaker implantation was performed for complete heart block in a patient with prior known metastatic melanoma to the brain with the incidental finding of a hypermetabolic cardiac mass on PET-CT [11]. To our knowledge, this is the first case of cardiac malignant melanoma presenting as a complete heart block at diagnosis. Pacemaker placement may help symptoms and provide support for the next phases of care, including surgical, radiation, and immuno-therapies. 

Transthoracic echocardiography is often the first imaging study performed but has limitations due to poor acoustic windows, poor resolution for posterior structures (intra-atrial septum, pulmonary veins, left atrial appendage, etc.), and limitations of tissue characteristics. Incidental findings on thoracic CT and MRI are also common as the first presentation of cardiac tumors. Additional advanced imaging is recommended to delineate size, evaluate surrounding structures, and determine if benign or malignant source of tumors.

Cardiac MRI provides detailed tissue characteristics and may narrow the differential diagnosis based on the presence or absence of LGE and T1- and T2-weighted imaging characteristics [12]. Metastatic melanoma has a high T1-weighted and low T2-weighted signal intensity, unlike other metastatic malignant tumors. Whole body positron emission tomography (PET) provides additional functional metabolic information, staging characteristics, and site of origin of metastatic tumors, and it is useful to assess response to therapy. 

Benign cardiac tumors that may mimic metastatic melanoma include rhabdomyomas, fibromas, lipomas, pericardial cysts, atrial myxoma, and hemangiomas [3]. Symptoms can occur due to bulky encroachment on the valve apparatus and due to micro-embolization and stroke. Systemic symptoms are rare. The mainstay of treatment is surgical resection without additional therapy, and long-term prognosis is favorable.

Treatment of malignant melanoma with metastatic disease to the heart includes chemo- and immunotherapy in conjunction with surgical treatment if anatomically feasible. If complete resection of all metastatic masses can be achieved, radiation therapy coupled with immunotherapy provides a survival benefit over surgery and radiation alone. In the absence of complete surgical resection, the overall prognosis is poor, with a mortality rate of over 60% at two years with standard therapy [5]. The presence of multiple metastatic sites is associated with the lowest survival rates [2].

## Conclusions

Cardiac metastatic melanoma is a rare condition with variable presentation. Cardiac symptoms, thromboembolic stroke, and abnormal findings on cardiac examinations should prompt a high index of suspicion. Advanced cardiac imaging with MRI and PET/CT are useful to confirm the diagnosis. The prognosis is guarded at the time of diagnosis, and survival is poor, especially in multi-organ involvement, despite advances in immunotherapy. 
